# Predicting the Influence of Early Trunk Control on Individual Functional Outcomes in Stroke Survivors at Three Months: A Longitudinal Observational Study

**DOI:** 10.3390/healthcare14183052

**Published:** 2026-09-17

**Authors:** Saeed Al Qahtani, Mashael Alsobhi, Fayaz Khan

**Affiliations:** Department of Physical Therapy, Faculty of Medical Rehabilitation Sciences, King Abdulaziz University, Jeddah 21589, Saudi Arabia; pt.saeed1@hotmail.com (S.A.Q.); mgalsobhi@kau.edu.sa (M.A.)

**Keywords:** stroke, activities of daily living, balance, community mobility, prediction, trunk control

## Abstract

**Background:** Stroke is a primary contributor to persistent disability. Trunk control is commonly disrupted after a stroke, and it is a critical motor skill for many static and dynamic functions that are affected post-stroke. **Objective:** This study set out to find out whether the activities of daily living ADLs, gait independence (FAC), falls, fear of fall, (MFES), balance (ABC), quality of life (SSQoL), and community mobility (CM) are predicted by using the Trunk Control Test (TCT) for stroke survivors after 3 months. **Methods:** Initially, sixty-three patients experiencing acute stroke were assessed to establish baseline measurements of stroke severity and trunk control. Subsequently, a follow-up was conducted after three months to assess ADLs, FAC, ABC, falls, MFES, SSQoL and CM to identify which variables were strongly predicted by trunk control. The data were analyzed using a multivariate general linear model (GLM) to predict the impact of TCT on each dependent variable. **Results:** The mean age of the subjects was 57.08 ± 14.81 years, with 55.6% (n = 35) being males. After adjustment for sex, age, time since onset, baseline severity and rehabilitation dose, TCT remained an independent predictor of all outcomes, BI: B = 0.75, bootstrap 95% CI 0.606 to 0.934; RNLI: B = 0.72, 95% CI 0.52 to 0.94; and MFES: B = 0.06, 95% CI 0.04 to 0.08 except for fall count. **Conclusions:** TCT is an early predictor of functional abilities and community participation among stroke survivors. It should be integrated into assessment and management processes during stroke rehabilitation.

## 1. Introduction

Stroke is recognized as the leading cause of chronic disability in adults [1]. The incidence of stroke is 29/100,000 annually in Saudi Arabia [2]. As the second leading cause of death, stroke also plays a significant role in contributing to disability, with ischemic strokes making up 62% of cases, intracerebral hemorrhages (ICHs) 28%, and subarachnoid hemorrhages (SAHs) 10% [3,4]. Following a stroke, there is often a notable impairment in trunk function, which adversely affects balance, gait, and activities of daily living (ADLs) [5,6]. Trunk control is an essential motor skill for many functions [7] involving the capacity of trunk muscles to sustain an upright posture, adjust to shifts in weight, and perform specific trunk movements while maintaining alignment over the base of support during both static and dynamic postural adjustments [8].

Post-stroke, bilateral deterioration of trunk muscles may occur, potentially resulting in an asymmetrical posture and hindering patients’ ability to support the affected side with their body weight [7]. Consequently, the patient’s capacity to maintain postural control and perform daily activities may be compromised following a stroke [9]. Identifying predictors of functional outcomes post-stroke has long posed a challenge in rehabilitation research [10]. Trunk function serves as a significant indicator of functional outcomes during stroke recovery [6,11,12]. Essential tasks, such as sitting, transitioning from a supine to a sitting position, and rolling, are heavily reliant on trunk function [13]. Therefore, it is crucial to identify outcomes that can be more accurately predicted by trunk control in stroke patients.

Trunk control is a critical component in the execution of activities of daily living (ADLs), as it enhances both mobility and postural stability. A recent study indicated that early trunk control in post-stroke patients serves as a predictor of ADL outcomes at later stages [13]. Additionally, trunk function has been shown to effectively predict ADLs when evaluated using the trunk impairment scale (TIS) as a measurement tool [13]. Furthermore, there is a significant correlation between trunk performance and balance, gait, and functional mobility [5]. Several studies have reported that trunk control is a crucial early predictor of gait independence in stroke patients [14,15,16]. Mahmood et al. also demonstrated that improved quality of life (QoL) outcomes are associated with enhanced trunk control three months post-stroke [17]. Moreover, trunk control significantly enhances patients’ ability to fulfill social obligations and integrate into the community, making it an essential component of early rehabilitation plans for stroke survivors [18].

Despite ongoing research, it remains uncertain which of these variables—ADL, gait independence, balance, falls, fear of fall, QoL, and CM—is most effectively predicted by trunk performance. Consequently, this study seeks to ascertain whether trunk function can serve as a predictor for ADL, gait independence, balance, falls, fear of fall, QoL, and CM in stroke survivors after a period of three months. The findings of this study will enable healthcare professionals, particularly physical therapists, to incorporate this outcome into early management strategies (within the initial three months) and enhance trunk performance to address limitations in this function.

## 2. Materials and Methods

### 2.1. Study Population and Sampling

This study utilized a longitudinal observational design, wherein patients were evaluated at baseline and subsequently reassessed three months later to ascertain the primary outcome predicted by trunk control among patients with stroke.

Patients with stroke were screened for eligibility based on specific inclusion and exclusion criteria. Subjects were included if they were clinically diagnosed with stroke within one month, confirmed by Computed Tomography (CT) or Magnetic Resonance Imaging (MRI), and were aged 18 years or older. Subjects with cognitive impairments (<22 in Montreal Cognitive Assessment), serious comorbid conditions, a brain stem stroke or bilateral stroke, and patients who were aphasic were excluded. Baseline stroke severity was quantified using the Modified Rankin Scale (mRS), a 6-point ordinal scale ranging from 0 (no symptoms) to 5 (severe disability); the National Institutes of Health Stroke Scale (NIHSS) was not administered in this cohort.

**Figure 1:**
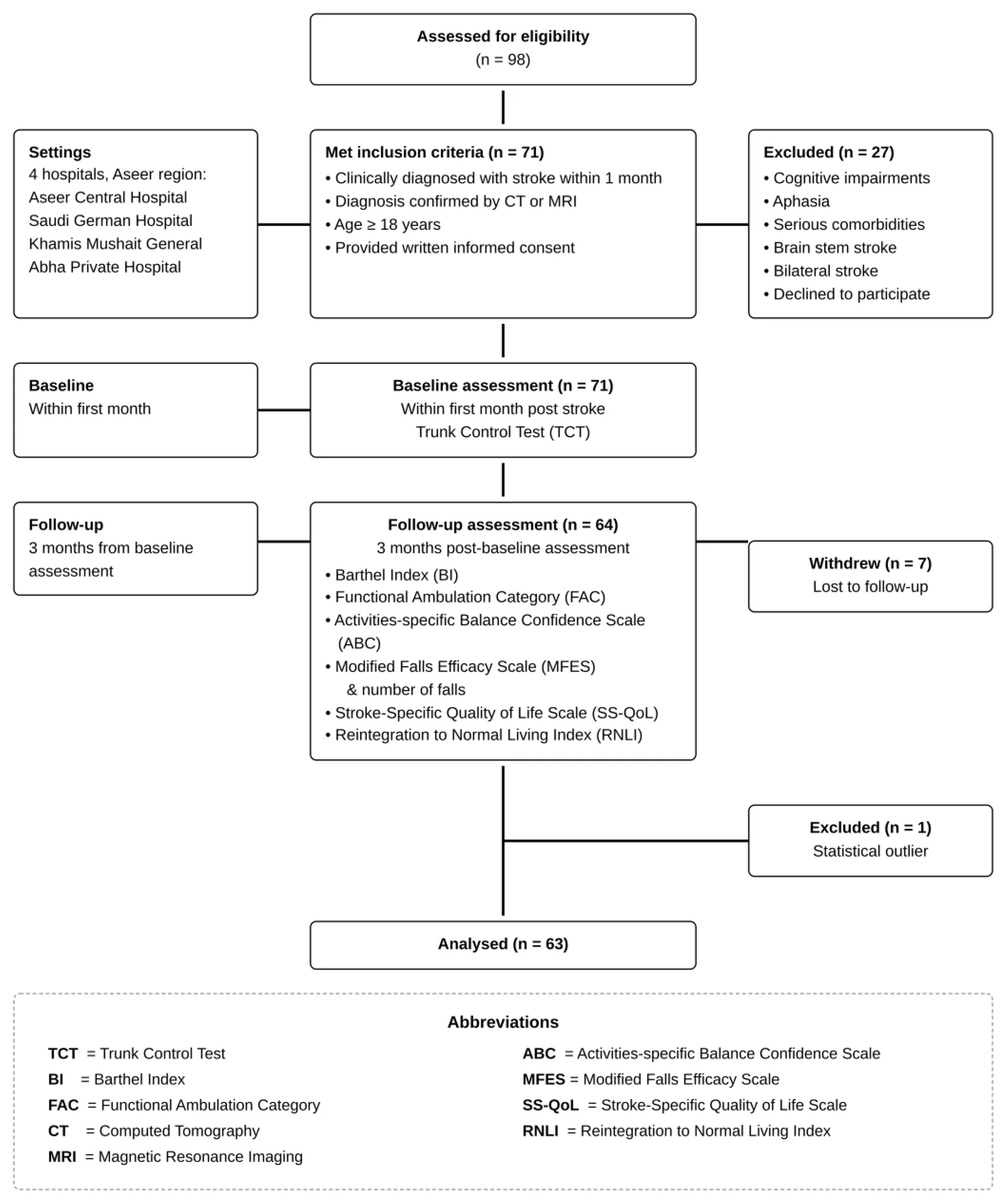
Participant flow diagram from screening to analysis.

Of the 98 patients assessed for eligibility, 27 did not meet the inclusion criteria. The remaining 71 participants met the inclusion criteria, 7 were subsequently lost to follow-up (Figure 1) and one patient was excluded in the analysis as an outlier leaving 63 participants assessed at three months with varying degrees of stroke severity, recruited through convenience sampling. Initial assessments were conducted using valid, reliable, and standardized measures for each variable of stroke severity and trunk control. Follow-up assessments were conducted after three months by the same assessor to evaluate ADLs, gait independence, balance, falls, QoL, and CM to identify which variables were strongly predicted by trunk control. Sociodemographic and clinical characteristics, including age, gender, type of stroke, hemiplegic side, and stroke duration, were documented. The sample size was determined using G*Power version 3.1, employing an F test for linear multiple regression. The parameters included an alpha level of 0.05, a power (1 − β) of 0.8, an effect size (f^2^) of 0.3, and seven predictor variables. The effect size of 0.3 was derived from a previous study examining the relationship between TCT and community mobility, which reported a standardized beta of 0.5, leading to the calculation of an effect size f^2^ of 0.3. Consequently, the anticipated sample size was calculated to be 56 participants [19].

### 2.2. Ethical Approval

Ethical approval was obtained from the institutional review board of the Ministry of Health, Aseer (IRB Log No: REC-07-10-2022), approved by the National Committee of Bioethics (Reg. No: H-06-B-091) dated 31 October 2022. Further ethical registration details are provided in the Institutional Review Board Statement. Written informed consent was obtained from all subjects, who were informed about the study objectives and protocol. Subjects were informed about the confidentiality of their participation and their right to withdraw at any time.

### 2.3. Data Collection

Following the receipt of ethical approval, data collection was conducted from November 2022 to October 2023 within the inpatient neurology departments of four major hospitals in Aseer, Saudi Arabia: Aseer Central Hospital, Khamis Mushait Hospital, Saudi German Hospital, and Abha Private Hospital. Upon discharge from these hospitals, the study participants were referred to the Saudi German Hospital in Abha, where a follow-up assessment was performed by SQ three months after the initial evaluation. All study participants received regular physical therapy for three months at the Saudi German Hospital.

### 2.4. Outcome Measures

#### 2.4.1. Independent Variable

The Trunk Control Test (TCT) was employed to evaluate trunk control, serving as a valid and reliable assessment instrument for individuals who have experienced a stroke. The TCT comprises four components, each assessed on a three-point ordinal scale: rolling to both the affected and unaffected sides, transitioning from a supine to a sitting position, and maintaining balance while sitting for 30 s. The total score ranges from 0 to 100 points, with higher scores indicating better performance [20].

#### 2.4.2. Dependent Variables

ADLs were evaluated using the Modified Barthel Index (BI) [21], which measures the individual’s performance on 10 ADL functions. It is an empirically derived scale that assesses an individual’s performance across ten ADL functions. This empirically derived scale has demonstrated inter-observer and test–retest reliability and validity, measuring a patient’s functional ability without the influence of family and social functioning [22].

Balance was assessed using the Arabic version of the Activity-Specific Balance Confidence (ABC) Scale [23]. The ABC scale is a self-assessment tool that measures an individual’s confidence in performing activities without losing balance. It is a highly sensitive and specific predictor of fall risk, with the highest possible score being 100%, indicating complete confidence in maintaining balance during various activities, from normal walking to walking on unstable surfaces [23].

Fear of falling was assessed using the Modified Fall Efficacy Scale (MFES). The MFES consists of 14 questions on specific activities and was used to assess patients’ confidence in independently completing the tasks. Patients rated each activity on a scale of 0 (not at all confident) to 10 (completely confident) and is evaluated by determining the mean score of all 14 completed items, each rated on a scale from 0 to 10 [24]. The frequency of falls was determined by conducting a monthly telephone interview with the patient, during which they were queried about the number of falls experienced in that month.

Gait independence was assessed using the Functional Ambulation Categories (FACs). The FACs is a reliable and valid instrument for classifying the level of physical support required by patients to ambulate safely post-stroke, encompassing six levels, ranging from 0, indicating “nonfunctional ambulation,” to 5, indicating “independent ambulation on level and non-level surfaces, including stairs and inclines” [25].

The evaluation of community reintegration was conducted using the 11-item Reintegration to Normal Living Index (RNLI/Arabic version). This index measures community participation, with a total score ranging from 0 to 110, derived from the sum of all 11 items [26]. The RNLI is a self-report measure consisting of 11 items related to the physical, social, and psychological aspects of daily life, such as mobility in the home and community, taking trips, self-care, work activities like volunteering, housework, studying, recreational activities, and social activities, as well as family role and perception-of-self items, including comfort with others, comfort with self, and ability to manage stress [26].

Quality of life (QoL) was evaluated using the Stroke-Specific Quality of Life Scale (SS-QOL/Arabic version). This scale comprises 12 categories and 49 questions, each graded on a scale from 1 to 5, covering domains such as mobility, upper limb functions, vision, social role, self-care, family role, work productivity, language, energy, mood, personality, and cognition. The total score is calculated by summing the item values; higher overall scores reflect a better health-related quality of life [27].

### 2.5. Statistical Analysis

The data were analyzed utilizing SPSS version 23 (SPSS, Inc., Chicago, IL, USA). Descriptive statistics were presented as frequency (n) and percentages (%) for categorical variables, and as mean ± standard deviation (SD) and median (minimum-maximum) for continuous variables. Simple linear regression was employed to assess the impact of TCT on each predictable variable. In accordance with the study’s objective, seven dependent variables and one independent variable were identified; consequently, a multivariate general linear model (GLM) with a bootstrap analysis was performed. Statistical significance was established at *p* ≤ 0.05.

## 3. Results

After adjustment for sex, age, time since onset, baseline severity and rehabilitation dose, TCT remained an independent predictor of all outcomes except fall count.

### 3.1. Demographic and Baseline Characteristics

The study analysis included a total of 63 participants, with a mean age of 57.08 ± 14.81 years. The gender distribution was nearly balanced, comprising 55.6% (n = 35) males and 44.4% (n = 28) females. The average time from stroke to recruitment was 10.34 ± 6.97 days. The baseline demographic characteristics of the participants are detailed in Table 1.

### 3.2. Simple Linear Regression

The findings from the simple linear regression analysis indicate that all variables exhibited a significant association with TCT, Barthel Index (B = 0.73, *p* ≤ 0.001, 95% CI; 0.65 to 0.81), fear of falling as measured by MFES (B = 0.06, *p* ≤ 0.001, 95% CI; 0.05 to 0.07), and all the other variables with a significant *p* ≤ 0.05. The associations between TCT and other predictive variables are detailed in Table 2.

### 3.3. Adjusted, Bootstrap-Validated Multivariate Analysis

After adjusting for sex, age, time since stroke onset, baseline Modified Rankin Scale score, and number of physiotherapy sessions received during follow-up in a multivariate general linear model, ADL measured using BI predicted using TCT (Barthel Index: B = 0.75, 95% CI 0.61 to 0.93), followed by community mobility (RNLI: B = 0.72, 95% CI 0.52 to 0.94), fear of fall (MFES: B = 0.06, 95% CI 0.04 to 0.08), ambulation (FAC: B = 0.03, 95% CI 0.02–0.05), balance confidence (ABC: B = 0.54, 95% CI 0.22–0.89) and quality of life (SSQOL: B = 0.58, 95% CI 0.29–0.94).

TCT was not significantly associated with the number of falls recorded during follow-up (B = 0.02, 95% CI −0.03 to 0.08). The R^2^ value was 0.85 and the adjusted R^2^ for the model was 0.83 (Table 3).

## 4. Discussion

This study investigated the extent to which trunk control predicts ADLs, gait independence, balance, falls, QoL, and CM in stroke patients after three months. The results indicated that all the six dependent variables were highly predicted by TCT except the number of falls. This finding aligns with previous research that identified trunk control as a significant predictor of ADLs, as measured by the Functional Independence Measure (FIM) at discharge [13]. Furthermore, prior studies have established that trunk control is crucial for functional movements and the coordination of upper and lower extremities necessary for daily living activities [5,7]. The finding that trunk control predicts ADL independence underscores the importance of early trunk rehabilitation interventions following a stroke.

Post-stroke unilateral trunk involvement results in impaired weight shifting towards the affected side, thereby disrupting balance in this population. Following a stroke, various factors, including spasticity and muscular and sensory deficits, may affect weight-shifting capabilities, potentially leading to balance impairments. The literature emphasizes the significance of balance in evaluating an individual’s walking abilities [5,27,28,29]. In this study, findings revealed that balance confidence and community mobility are also well predicted by trunk control, corroborating existing evidence that highlights the beneficial impact of trunk rehabilitation on balance and mobility in stroke patients. Additionally, previous research has demonstrated a correlation between trunk flexion and extension muscle weakness and the Berg Balance Scale [30]. Furthermore, a review study indicated that trunk training can enhance trunk control, sitting and standing balance, and mobility among stroke patients [31].

The impairment of trunk function is a critical factor contributing to the abnormal gait patterns observed following a stroke [32,33]. Tsuji et al. identified correlations between trunk function, FIM scores, and gait speed, which align with the findings of this study, indicating a significant association between TCT and FAC [34]. Previous research has demonstrated that reduced gait speed, unequal steps, and limited endurance are correlated with diminished trunk control [5,32]. Based on these findings, trunk control can serve as a prognostic indicator and rehabilitation goal for enhancing gait in stroke patients. Furthermore, the results suggest that TCT scores at stroke onset are predictive of balance and mobility in later stages. These findings corroborate the results reported by Van Criekinge et al., who provided strong evidence for the effectiveness of trunk training in improving balance and mobility in patients with stroke [31].

In relation to fear of fall, this study demonstrates that TCT can predict the fear of falling, aligning with previous research indicating that trunk control influences fear of falling in patients with stroke [35]. This finding is also consistent with the study by Khallaf et al., which concluded that inadequate trunk control is associated with falls in patients with stroke [36]. Similarly, impaired trunk function has been linked to postural instability, mobility challenges, and confidence issues [5]. The predictive relationship between trunk control and fear of falling suggests that incorporating trunk interventions in early stroke rehabilitation may reduce the risk of falls. Post-stroke recovery of trunk function has been associated with the compensatory activation of uncrossed pathways [37]. The results of the current study indicate that fall count was not a significant predictor within the model. However, as an individual predictor, it was shown to be significant. This discrepancy may be attributed to other physical factors, such as muscle strength during the acute stage of stroke, which could have influenced these outcomes.

In our cohort, better early trunk control, as assessed by the Trunk Control Test (TCT), was associated with higher Stroke-Specific Quality of Life (SSQOL) scores at three months, suggesting that proximal postural control may contribute meaningfully to stroke survivors’ perceived health status and participation. This finding is consistent with prior work demonstrating that trunk performance is closely related to balance, gait, functional independence, and broader community reintegration after stroke, domains that are also captured within stroke-specific quality of life instruments [19]. In particular, studies using detailed trunk performance measures have reported that impaired trunk control is linked to limitations in activities of daily living and reduced participation, which in turn have been identified as key determinants of poorer quality of life in subacute and chronic stroke populations [38]. The SSQOL itself was developed to capture these patient-centered dimensions of recovery, including mobility, self-care, social roles, mood, and thinking, all of which may be indirectly influenced by the ability to maintain and adjust trunk posture during everyday tasks [39]. While our observational design precludes causal inference, the observed association between higher early TCT scores and better SSQOL outcomes supports the view that trunk control represents a relevant prognostic domain for health-related quality of life and reinforces calls to systematically assess and address trunk performance within comprehensive stroke rehabilitation programs.

The lateral motor control system, including the lateral corticospinal tract and rubrospinal tract, regulates distal limb movements, finger motor movements, and sensory input related to the paralyzed side and trunk function. The trunk and proximal muscles involved in standing, walking, postural reflexes, parallel function, and muscle tone are regulated by the medial motor control system, which comprises the corticospinal tract, lateral vestibulospinal tract, reticulospinal and tectospinal tracts, and anterior corticospinal tract [40,41]. Daily functioning cannot be achieved if the internal motor control system is compromised, even when only the external motor control system is affected, due to the fundamental role of postural control [13]. Therefore, the recovery of trunk function is biomechanically and neurophysiologically crucial for regaining motor control and mitigating psychological issues associated with the fear of falling.

This study has several limitations that must be acknowledged. Firstly, the longitudinal design, which included two consecutive time points (baseline and three months), may not comprehensively capture the entire trajectory of functional recovery. Secondly, a key limitation of this study is the absence of performance-based outcome measures, particularly for balance, as we relied primarily on patient-reported scales such as the Activity-Specific Balance Confidence (ABC) Scale. Self-report instruments are susceptible to recall bias, response bias, and individual differences in risk perception, which may not accurately reflect actual balance capacity or functional performance. Consequently, our findings regarding the influence of early trunk control on balance and related functional outcomes should be interpreted with caution, as they may over- or underestimate the true association. Lastly, the cohort analyzed was drawn from a single geographical region and consisted exclusively of ischemic stroke cases after exclusions, limiting the generalizability of the findings to hemorrhagic stroke and other settings.

## 5. Conclusions

In conclusion, this study elucidates the factors most accurately predicted by trunk function. These findings hold significant value for rehabilitation professionals, as they can inform policy development, the establishment of comprehensive rehabilitation programs, and the prioritization of trunk control exercises during the early stages of rehabilitation to maximize patient independence. The study posits that trunk control serves as a dynamic predictor of functional independence following a stroke. Future research is recommended to compare the influence of the upper and lower extremities and the trunk on these variables to validate the study’s results. Consequently, tailoring trunk-specific training may become central to early rehabilitation programs for stroke patients, enhancing overall quality of life, balance, and mobility. This conclusion underscores the need to revise clinical protocols to incorporate trunk exercises as a fundamental component of post-stroke recovery strategies.

## Figures and Tables

**Table 1:** Demographic characteristics of the participants.

Descriptive Statistics (n = 63)	
Variables	Frequency (%)
Gender (Male/Female)	35 (55.6) 28 (44.4)
Fall (Fallers/Non-Fallers)	33 (52.4) 30 (47.6)
Hemiplegic Side (Right/Left)	31 (49.2) 32 (50.8)
	**Mean ± SD (Range)**
Age	57.08 ± 14.81 (24 to 95)
Stroke Duration (Days)	10.34 ± 6.97 (2 to 28)
	**Mean ± SD; Median (Range)**
TCT	51.28 ± 38.06; 50 (0 to 100)
mRS	3.58 ± 1.29; 4 (1 to 5)
FAC	2.82 ± 1.79; 3 (0 to 5)
ABC	53.91 ± 30.90; 53 (6.25 to 98.75)
BI	66.58 ± 30.02; 85 (25 to 100)
RNLI	63.04 ± 24.71; 68.18 (9.60 to 98.10)
SSQOL	172.88 ± 34.32; 179 (98 to 231)
MFES	4.95 ± 2.79; 4.64 (0.57 to 9.80)
PT Sessions During Follow-Up	25.03 ± 17.27; 24 (0 to 65)
No. of Falls	2.49 ± 3.92; 1 (0 to 20)

**Table 2:** Simple linear regression for study variables.

	Unstandardized Coefficients		Standardized Coefficients			95% CI	
Model	B	Std. Error	Beta	t	*p*	Lower Bound	Upper Bound
FAC	0.04	0.003	0.84	11.88	≤0.001	0.03	0.05
ABC	0.69	0.05	0.85	12.42	≤0.001	0.57	0.79
BI	0.73	0.04	0.92	18.70	≤0.001	0.65	0.81
RNLI	0.53	0.05	0.82	11.14	≤0.001	0.43	0.63
SSQOL	0.69	0.08	0.76	9.23	≤0.001	0.54	0.84
MFES	0.06	0.01	0.87	13.79	≤0.001	0.05	0.07
Number of Falls	0.02	0.01	0.26	2.16	0.03	0.002	0.05

Abbreviations: FACs, Functional Ambulatory Categories; ABC, Activity-Specific Balance Confidence Scale; BI, Barthel Index; RNLI, Reintegration to Normal Living Index; SSQOL, Stroke-Specific Quality of Life; MFES, Modified Fall Efficacy Scale.

**Table 3:** Multivariate GLM between TCT and 3-month functional outcomes.

Outcome	B	Bootstrap SE	Bootstrap 95% CI	Sig.	Partial η^2^ (TCT)	R^2^	Adjusted R^2^
FAC	0.03	0.01	0.02 to 0.05	≤0.001	0.26	0.72	0.69
ABC	0.54	0.17	0.22 to 0.89	≤0.001	0.26	0.75	0.72
BI	0.75	0.08	0.61 to 0.93	≤0.001	0.56	0.85	0.84
RNLI	0.72	0.10	0.52 to 0.94	≤0.001	0.47	0.72	0.68
SSQOL	0.58	0.17	0.29 to 0.94	≤0.001	0.17	0.59	0.55
Number of Falls	0.02	0.03	−0.03 to 0.08	0.366	0.01	0.15	0.06
MFES	0.06	0.01	0.04 to 0.08	≤0.001	0.39	0.78	0.75

Abbreviations: FACs, Functional Ambulatory Categories; ABC, Activity-Specific Balance Confidence Scale; BI, Barthel Index; RNLI, Reintegration to Normal Living Index; SSQOL, Stroke-Specific Quality of Life; MFES, Modified Fall Efficacy Scale.

## Data Availability

The data presented in this study are available on request from the corresponding author. The data are not publicly available due to ethical restrictions.
